# Prediction of Toxin Genes from Chinese Yellow Catfish Based on Transcriptomic and Proteomic Sequencing

**DOI:** 10.3390/ijms17040556

**Published:** 2016-04-13

**Authors:** Bing Xie, Xiaofeng Li, Zhilong Lin, Zhiqiang Ruan, Min Wang, Jie Liu, Ting Tong, Jia Li, Yu Huang, Bo Wen, Ying Sun, Qiong Shi

**Affiliations:** 1 Section on Marine biobank, China National Genebank, Shenzhen 518083, China; xiebing@genomics.cn (B.X.); bgistone@163.com (X.L.); 2 BGI-Shenzhen, Shenzhen 518083, China; linzhilong@genomics.cn (Z.L.); liujie8@genomics.cn (J.L.); tongting@genomics.cn (T.T.); 3 Shenzhen Key Lab of Marine Genomics, Guangdong Provincial Key Lab of Molecular Breeding in Marine Economic Animals, Shenzhen 518083, China; ruanzhiqiang@genomics.cn (Z.R.); wangmin2@genomics.cn (M.W.); lijia1@genomics.cn (J.L.); huangyu@genomics.cn (Y.H.); 4 Center for Marine Research, College of Life Sciences, Shenzhen University, Shenzhen 518060, China

**Keywords:** Chinese yellow catfish, venom, transcriptome, proteome

## Abstract

Fish venom remains a virtually untapped resource. There are so few fish toxin sequences for reference, which increases the difficulty to study toxins from venomous fish and to develop efficient and fast methods to dig out toxin genes or proteins. Here, we utilized Chinese yellow catfish (*Pelteobagrus fulvidraco*) as our research object, since it is a representative species in Siluriformes with its venom glands embedded in the pectoral and dorsal fins. In this study, we set up an in-house toxin database and a novel toxin-discovering protocol to dig out precise toxin genes by combination of transcriptomic and proteomic sequencing. Finally, we obtained 15 putative toxin proteins distributed in five groups, namely *Veficolin*, *Ink toxin*, *Adamalysin*, *Za2G* and *CRISP* toxin. It seems that we have developed a novel bioinformatics method, through which we could identify toxin proteins with high confidence. Meanwhile, these toxins can also be useful for comparative studies in other fish and development of potential drugs.

## 1. Introduction

From an evolutionary perspective, venom, a complicated biochemical compound of biologically active components, such as polyamines, peptides, amino acids, neurotransmitters and proteins, has consolidated diversification and has been the successful survival of venomous organisms, such as scorpions, spiders, and lionfish [1]. Venom would be able to be injected by using some specialized apparatus to subdue prey or defend against predators [2]. Many previous studies focused on potential pharmacological agents or physiological tools on the basis of development of toxins from some terrestrial venomous animals, such as centipedes, snakes, scorpions, and spiders. Meanwhile, some pharmaceuticals with clear function derived from snake venoms, such as anticoagulation and antiangiogenesis, have already been developed for drugs [3]. In contrast, previous studies did not provide more interesting investigations based on venomous fish. Some recent studies roughly provided a dozen toxins that have been identified and characterized from several venomous fish [4], but it would just be the tip of the iceberg, considering the number of given venomous fish (~200 species) [2]. A complete understanding of the venomous fish and their venom would be significantly valuable for developing novel and effective therapeutic agents.

Traditional strategies for venom analysis are typically involved with bioassay-guided fractionation, where fractions displaying the desired biological activity in a specific assay are further investigated by purification and characterization [5]. However, this approach is time consuming and requires large amounts of crude venom.

Since the beginning of the 21st century, the next-generation sequencing (NGS) and liquid chromatography-tandem mass spectrometry (LC-MS/MS) technologies have greatly promoted the multi-omics study in a more sensitive and efficient approach with lower cost and lead to wide research of venom genes coming true [1,3], which has been proven successful in several domains such as neuroendocrine research and biomarker or drug discovery for medical areas [6]. What is more, the utilization of *de novo* assembling algorithms for deep sequencing has made it come true and was widely used in large-scale genomics and transcriptomics sequencing projects, with the accurate assembly of fragment data without reference genome into full-length transcripts in particular in the absence of a reference genome sequence [7]. A strategy integrating transcriptomic and proteomic/peptidomic approaches using bioinformatics can reveal “deep venomics” [8].

Our current study focused on analysis of transcriptome and proteome from a venomous teleost representative, Chinese yellow catfish (*Pelteobagrus fulvidraco*), which is one of the virulent bony fish in fresh water and is widespread in China [8]. According to some Chinese publications in the 1980s, there are venom glands in the spines in its dorsal fins and pectoral fins. Once stuck by those spines, the poison can cause excruciating local pain, edema, bleeding, and even lead to fever.

**Figure 1:**
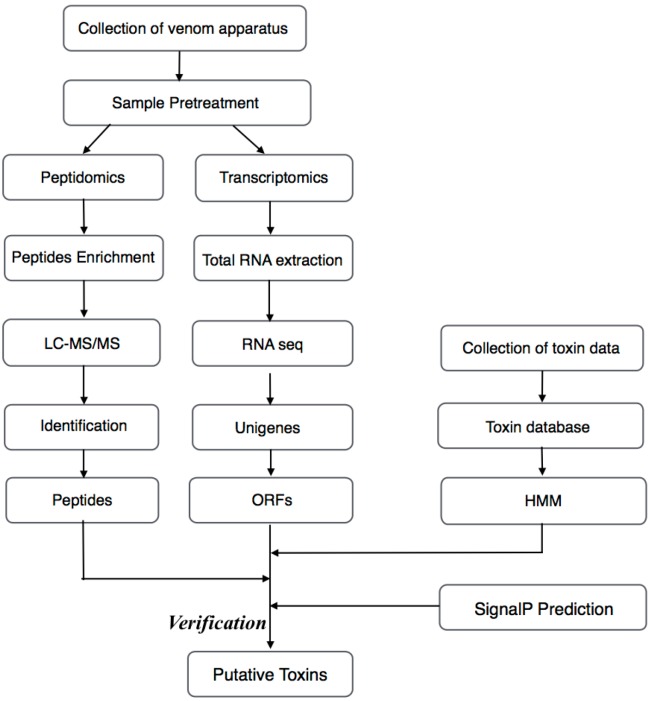
Schematic workflow for the transcriptomics and peptidomics analyses of the venom from the Chinese yellow catfish. ORFs, open reading frames; SignalP, signal peptides.

In this study, we provide not only the data of its transcriptome and proteome for the subsequent multi-omics analysis, but also a novel multi-omics pipeline (Figure 1) and a comprehensive database Hidden Markov Models (HMM) for the excavation of fish venoms, which can also be used for reference for the venom study of other venomous animals. The overall integration of such results will allow the computational simulation of many aspects of the complex biological interactions influencing the evolution and adaptation of venom genes, which can be validated by experimental functional assays. The evolutionary significance of relevant mutations may be of particular interest for human health and pharmaceutical research and could lead to the production of more efficient toxin antidotes. Moreover, understanding the genetic basis of the diversification of venom-encoding genes across various groups of ray-finned fish can provide fundamental biological insights into species evolution, ecological specialization, genetic novelties and drug developments, which may be of major importance for evolutionary and biomedical research.

## 2. Results and Discussion

### 2.1. LC-MS/MS Data Analysis

The raw LC-MS/MS data were converted into Mascot generic format (MGF) by Proteome Discoverer 1.3 (Thermo Fisher Scientific, Waltham, MA, USA). The RNA-Seq sequences were translated into protein sequences as the searching database. The MS/MS spectra were searched by Mascot (v2.3.02, MatrixScience) (Matrixscience, Boston, MA, USA) against the achieved protein database. The main parameters were set as follows: no specific enzyme digestion; Carbamidomethyl (C) as fixed modification; Oxidation (M) and Gln- > pyro-Glu (N-term Q) as variable modifications; peptide mass tolerance, 10 ppm; MS/MS tolerance, 0.02 Da; peptide charge, 2+, 3+ and 4+. The target-decoy search strategy was used to control confidence of peptide identification with False Discovery Rate (FDR) less than 1% [9,10]. Finally, 32,281 spectra were produced from the MS/MS data; 1240 peptides were identified in the further database search, mapping to 453 proteins after elimination of redundancy.

### 2.2. Assembly and ORF Prediction

A total of 44,243,514 raw reads were obtained through the Illumina HiSeq™ 2000 platform (Illumina, San Diego, CA, USA). After cleaning and removing those dirty reads containing adapters, unknown or low quality bases, we generated a total of 39,943,854 clean reads corresponding to more than 3.59 billion clean nucleotides. The average length of the clean reads was 90 bp, consistent with the sequencing capacity of the Illumina device. The Q20 percentage, N percentage and GC percentage were 96.12%, 0.31% and 46.11%, respectively. The original sequencing data for the clean reads have been submitted to the NCBI Sequence Read Archive (SRA) database (accession number SRP057554).

**Figure 2:**
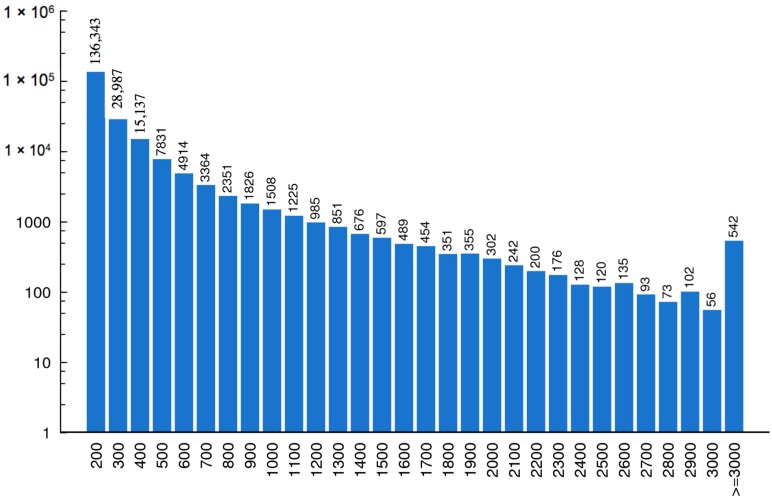
Length distribution of contigs. The horizontal coordinate represents contig length and the vertical coordinates stand for the number of contigs.

**Figure 3:**
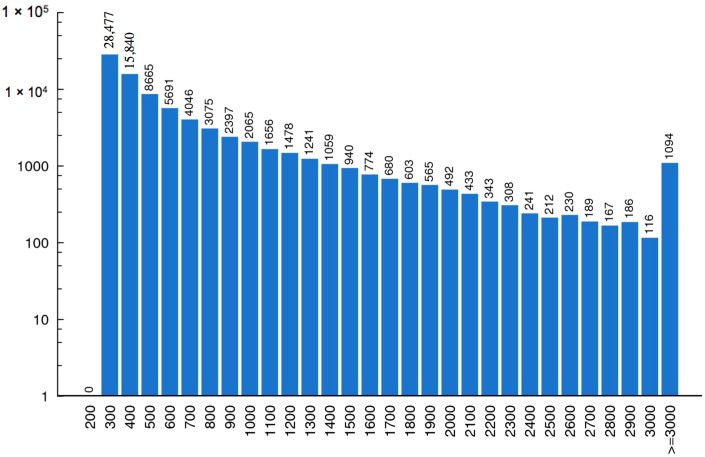
Length distribution of unigenes. The horizontal coordinate represents unigene length and the vertical coordinate stands for number of unigenes.

With running the Trinity [11] program, a total of 210,413 contigs corresponding to more than 57 million nucleotides were assembled from the short reads. Finally, the contigs were connected, and 83,263 unigenes were generated, with a mean length of 619 nucleotides. The length distributions of these assembled contigs and unigenes are shown in Figure 2 and Figure 3.

We got open reading frames (ORFs) from the assembled unigenes by ORFcor [12] with the reference of 12 fish genomes (downloaded from Ensemble), namely *Astyanax mexicanus* (GCA_000372685.1), *Danio rerio* (GCF_000002035.4), *Gadus morhua* (GCA_000231765.1), *Gasterosteus aculeatus* (GCA_000180675.1), *Lepisosteus oculatus* (GCF_000242695.1), *Oreochromis niloticus* (GCF_000188235.2), *Oryzias latipes* (GCF_000313675.1), *Petromyzon marinus* (GCA_000148955.1), *Takifugu rubripes* (GCF_000180615.1), *Tetraodon nigroviridis* (GCA_000180735.1), *Xiphophorus maculatus* (GCF_000241075.1), *Scleropages Formosus* (LGSG1000000). From the original 83,265 unigenes, we predicted 31,358 unigenes with ORFs.

### 2.3. Construction of Toxin Database

After a quick and simple survey of publications, we found that, unlike venom animals like scorpions, spiders and snakes, there is no special toxin database or dataset for excavation of fish toxins. Despite databases such as NCBI-RefSeq, NCBI-nucleotide, UniProtKB/Swiss-Prot and TrEMBL including all toxin sequences, there are more non-toxin sequences than toxin sequences. It will always be redundant and time-consuming for the alignment job. Hence, we need a special and comprehensive dataset as the reference. Among all the public toxin databases, we noticed that the animal toxin annotation program [13] is the latest update and systematically targets annotates of animal venom genes and proteins. The previous reported venomous animals usually include snakes, spiders, scorpions, cone snails, jellyfish, sea anemones, lizards, a few fish and platypuses. The program also provides us with the manual annotation of toxins produced by poisonous animals that lack venom injection devices, such as toads, ticks and worms. The number of total reviewed venom protein entries is 6058. Venom protein in UniprotKB/Swiss-Prot distributed by targets types: ion channels, receptors (7TM and 1TM) and transporters, and there is more detailed distribution in each of those four target types. In total, there are 27 groups.

It has also inspired us a lot in building an appropriate for our study. We collected toxin sequences from different databases as much as possible so as to build our in-house toxin database. Most of the amino acid and nucleotide sequences of toxins were retrieved from public databases such as the UniProtKB/Swiss-Prot and TrEMBL, NCBI-RefSeq, NCBI-nucleotide, and from certain special toxin databases including the Tox-Prot program [14], ConoServer [15], Animal Toxin Database [16], and ArachnoServer [17]. All these databases are very famous and extensively cited in toxin researches, but the toxin sequences in these databases are illustrated in different titles and formats. Therefore, we aligned these sequences with those sequences from NCBI and set their information in a unified criterion for construction of our database. First, we downloaded sequences with keywords *Toxin* and *Venom* according to the *Taxonomy* from NCBI, and then we screened out those sequences with keywords *Predicted*, *Hypothetical*, *Putative*, *Unnamed*, *Uncharacterised*. Second, we checked the left sequences manually based on sequence and species information with the following curation [18]: 1. Venom protein names and synonyms; 2. Functional annotation with use of current nomenclatures; 3. Sequence feature annotation; 4. Literature annotation. Finally, a total of 8863 toxin or venom sequences were retrieved (Table 1).

### 2.4. Excavation of Toxin Genes

In previous studies of venom animals [1,8], *Blast* [19] is the most popular alignment tool for checking the homologies of toxin genes. We did utilize *Blast* at first, but the outcome showed that the homologous loci are distributed dispersedly rather than a centered distribution. As for these homologous loci, we have no idea whether they are vital functional or conservative for the toxic reaction, and the number of the so-called homologous sequences is so large that it is a vast project for us to refer to relative documents. The situation is the same with the other three most used alignment programs, namely *PsiBlast* [20], *Phmmer* and *Jackhmmer* [21]. As we mentioned above, there are so few fish toxin sequences for reference and the other venom species are long distant distributed; therefore, it is no wonder that the alignment results are barely satisfied.

Fry [22] looked deep into the origin and evolution of snake venom proteome by means of phylogenetic analysis of the amino acid sequences of the venom proteins and their orthological non-venom proteins, and demonstrated that the snake toxins have originated from those genes which distribute in different non-venom tissues in the recruitment events. These toxin proteins and their homologous body proteins can also be found in many other distant species, such as cone snail, fire ant, cat flea, stable fly, yellow mosquito, giant honeybee, and even rats and humans. However, modifications have taken place diversely in these distant species. Therefore, it is hardly to find out orthologs in our fish venoms via the commonly used alignment tool *Blast*. In order to cut down the difficulty from the diverse phylogenetic recruitments to look for orthologous toxin sequences, we utilized the method of building Hidden Markov Models (HMMs) from toxin sequences of different types to search for toxin transcripts in our fish venom. We came up with a novel bioinformatics analysis protocol by combing transcriptomics with proteomics. To build HMMs, first we searched our in-house database with Jackhmmer (default cutoff *e*-value of 10^−5^), and sequences were clustered into 138 groups according to their conservative loci and sequence patterns. Subsequently, we combined these 138 groups and those 27 groups clustered from the websites. Finally, we obtained 165 groups of toxin sequences for further multi-alignment of the sequences in each group with MsaProbs [23] and HMM model analysis by HMM Build. These achieved 165 HMM models were searched using HMM search (default cutoff *e*-value of 10^−4^) against the generated 31,358 unigenes, and 1151 sequences were hit.

**Figure 4:**
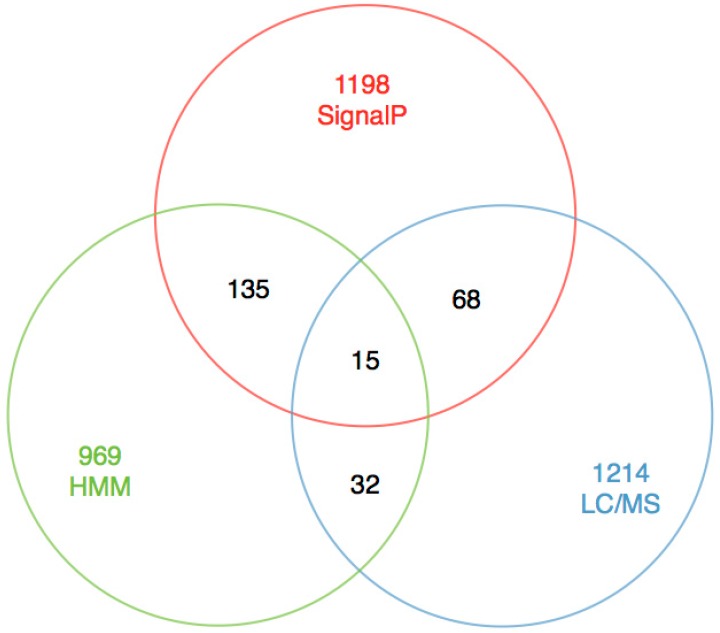
The statistics for SignalP, HMM search, LC-MS/MS and their mutual overlaps.

The main objective of this study is to obtain precise toxin sequences rather than the number of toxins. We tried to perform signal peptide prediction with SignalP 4.0 [24], since signal peptide is the most conserved component of toxin precursors [16], and 1416 unigenes out of these 31,358 unigenes were picked out. These sequences were clustered into 780 groups according to their similarities in sequence pattern and function by Jackhmmer (default cutoff *e*-value of 10^−5^). After removing the redundancy, we obtained 15 sequences (DNA sequences in Supplementary S1 and protein sequences in Supplementary S2) with hits from the LC-MS/MS sequencing (Figure 4).

We also learnt that not all sequences get their signal peptides. One common situation is that signal peptides are eliminated while toxins are being transported through the cell membrane and forming to mature proteins. The other common reason is that the signal peptides got lost since the assembly technique is not so perfect at the present time [25]. It is no denying that there are still some toxin proteins in the overlap of HMM and LC-MS/MS despite their signal peptides were not predicted. For this part, we will carry out more detailed analysis in future research since we have several more venom fish sequenced.

The significant homology sequences of these 15 transcripts were also found in the genome of *Scleropages formosus*, *Salmo sala*, *Astyanax mexicanus* and *Callorhinchur milii* with Blast (*e*-value 10^−5^), indicating the theory by Fry [22] is correct and our HMMs are also built upon correct hypothesis. We do not deny that this pipeline is very strict for screening out putative toxin proteins, and these new sequences are waiting for further functional assays. Despite the few sequences, these confident toxin sequences will set up a window for us to explore more from the venom of Chinese yellow catfish and other venomous fish.

### 2.5. Toxin Classification

After a series bioinformatical analysis, we identified several protein types distributed in five different superfamilies in the venom tissue of Chinese yellow catfish, namely Veficolin, Ink Toxin, Adamalysin, Zn-α2-Glycoprotein (ZaGP) and Cysteine-Rich Secretory Proteins (CRISP).

#### 2.5.1. Veficolin

**Figure 5:**
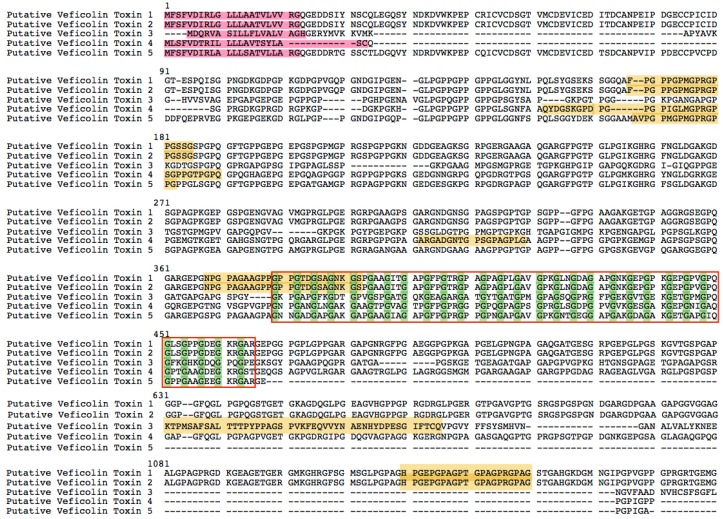
The putative *Veficolin* toxin sequences from Chinese yellow catfish. Sequences in red background are signal peptides. Peptides in yellow background are verified by LC-MS/MS analysis. The amino acid *Glycine* are marked in green color. The G-X-Y repeats are included in the red box. Dashed lines means the absence of corresponding amino acid sequences.

We found five transcripts (Figure 5) homologous to the Veficolin toxin sequences (homologies’ alignments in Supplementary S3). Veficolin was first identified in snake venom and then Anguimorpha lizard, but its function was not clearly characterized [1]. We find a domain contains large numbers of G-X-Y repeats: an alignment contains 20 copies of the G-X-Y repeat that form a triple helix [1]. The first position of the repeat is glycine, the second and third positions can be any residue but are frequently proline and hydroxyproline. Domains with this pattern are post translationally modified by proline hydroxylase to form the hydroxyproline residues, and defective hydroxylation is the cause of scurvy [26]. Thus, we infer that the veficolin might cause scurvy as well, which is in accordance with the symptoms caused by getting stuck by those spines from the Chinese yellow catfish.

#### 2.5.2. Ink Toxins

**Figure 6:**
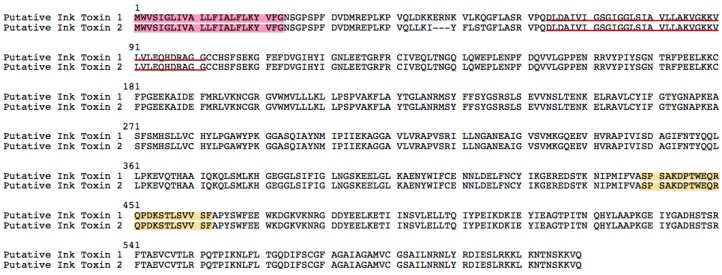
The putative Ink toxin sequences from Chinese yellow catfish. Sequences in the red background are signal peptides. Peptides in the yellow background are verified by LC-MS/MS analysis. The red underlined regions are the FAD domains.

Two transcripts (Figure 6) were found homologous to Ink toxin (homologies’ alignments in Supplementary S4). Ink toxin is a toxin protein, and it has been reported that several proteins, isolated from purple ink secretions of sea hares can act as antimicrobial and antitumor agents [27,28,29]. While being exposed to the Ink, tumor cells exhibited shrinkage of nuclei, loss of contact to adjacent cells and appearance of vacuoles in the endochylema [30]. This kills tumor cells within 6–8 h in an apoptosis independent manner by production of high amounts of hydrogen peroxide, which induces a necrotic form of oxidative stress; subsequent sequencing of the Ink toxin revealed a common flavin adenine dinucleotide (FAD)-binding domain [31].

#### 2.5.3. Adamalysin

**Figure 7:**
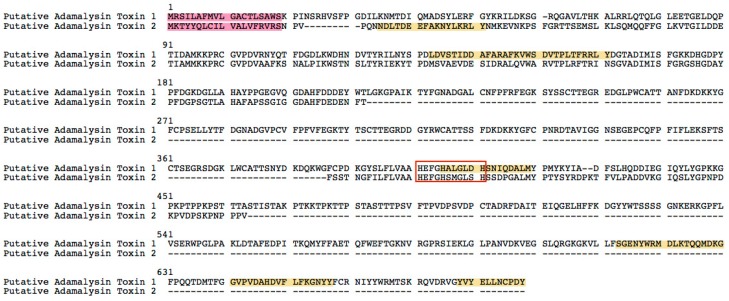
The putative *Adamalysin* toxin sequences from Chinese yellow catfish. Sequences in red background are signal peptides. Peptides in yellow background are verified by LC-MS/MS analysis. The red box includes the elongated zinc binding consensus sequence HEXXHXXGXXH. Dashed lines means the absence of corresponding amino acid sequences.

Two transcripts (Figure 7) encoding Adamalysin were identified in our transcriotome data (homologies’ alignments in Supplementary S5). Adamalysin, which requires zinc and calcium for its activity, is a toxin protein firstly isolated from the venom of Eastern diamond back rattlesnake (*Crotalus adamanteus*) [32]. The adamalysins comprise an elongated zinc binding consensus sequence HEXXHXXGXXH and a so-called “Met-turn” forming the hydrophobic base of the catalytic zinc-binding site. The three histidine residues of the consensus sequence are involved in zinc ligation, and the glutamic acid residue presumably is the general base in catalysis [33,34].

#### 2.5.4. Zn-α2-Glycoprotein

**Figure 8:**
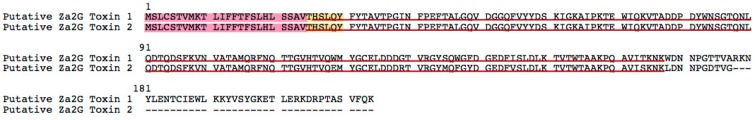
The putative *Za2G* toxin sequences from Chinese yellow catfish. Sequences in red background are signal peptides. Peptides in yellow background are verified by LC-MS/MS analysis. The red underlined domain are highly homologous to the MHC_I superfamily. Dashed lines means the absence of corresponding amino acid sequences.

Two transcripts (Figure 8) encoding Zinc-α2-glycoprotein (ZaGP) were identified (homologies’ alignments in Supplementary S6). ZaGP has been reported involved in both inhibition of tumor growth and proliferation, and its structure and sequence are highly homologous to a major histocompatibility complex class I (MHC_I) superfamily, which may function importantly in immunity [35], and ZaGP may have some protective effects in tumor patients and prevent the cancer progression [36].

#### 2.5.5. Cysteine-Rich Secretory Proteins Toxin (CRISP)

**Figure 9:**
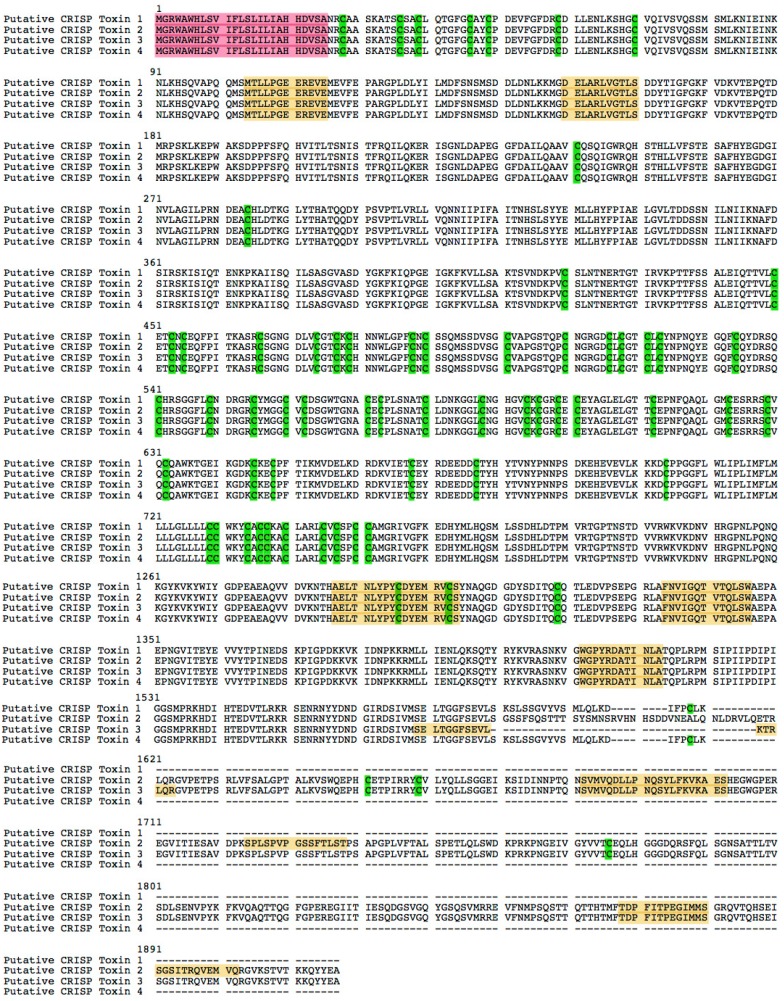
The putative *CRISP* toxin sequences from Chinese yellow catfish. Sequences in red background are signal peptides. Peptides in yellow background are verified by LC-MS/MS analysis. The amino acid *Cysteine* is marked in green. Dashed lines means the absence of corresponding amino acid sequences.

Four transcripts (Figure 9) are similar to CRISP toxins according to cysteine numbers and structure (homologies’ alignments in Supplementary S7). Recent research has revealed that CRISPs are widely distributed in snake venom and that they inhibit smooth muscle contraction and cyclic nucleotide-gated ion channels, block voltage-gated calcium and potassium channels and ryanodine receptors producing lethargy, paralysis, and hypothermia [29,37].

### 2.6. Verification

We carried out RT-PCR to verify these 15 putative toxin proteins. Since some sequences are so long that it is not easy to amplify the whole sequences, we sliced the whole sequence with random slices about 600–700 bp long. Then, we amplified these splices and verified them. All these sequences were confirmed (Supplementary Figure S1).

## 3. Materials and Methods

### 3.1. Specimens

Chinese yellow catfish was purchased in a local market in Yantian District, Shenzhen, China. All samples were verified by DNA barcoding. Spine samples were collected under the permit of the Institutional Review Board on Bioethics and Biosafety of BGI (No. FT 15048) and immediately snap frozen in liquid nitrogen then stored at −80 °C until future usage. Ten fish spines were collected for LC-MS/MS sequencing and fourteen fish spines for RNA-seq.

### 3.2. MS

#### 3.2.1. Venom Sample Preparation

The venom was denatured with 8 M urea in 0.1 M Tris-HCl, pH 8.5 [38] and the concentrations were resolved with the Bradford reagent (Sigma, St. Louis, MO, USA) and BSA (Sigma, St. Louis, MO, USA) as a standard protocol [39]. SDS-PAGE was performed according to Laemmli [40]. In brief, denatured venom was reduced with 10 mM Dithiothreitol (DTT) at 56 °C for 1 h. After being cooled down to room temperature, the venoms were alkylated with 55 mM Iodoacetamide (IAM) in the dark at room temperature for 45 min [41]. The alkylated venom solution was diluted with 8 M urea to 1 mL, and then fractionated in Strata-X C18 column (Phenomenex, Torrance, CA, USA), which has previously been conditioned with methanol. After loading the venom solution, the column was washed with 0.1% formic acid (FA) in 5% acetonitrile (ACN) and eluted with 80% ACN. The eluates were dried in a SCANVAC (Denmark) concentrator (LaboGene, Lynge, Denmark) and then stored at −20 °C for further analysis. After dissolved in 0.1% FA, the venom concentrations were determined using a nanodrop system (Thermo Scientific, Waltham, MA, USA).

#### 3.2.2. Nano LC-MS/MS Analysis

LC-MS/MS were performed on a prominence nano-HPLC system (Shimadzu, Tokyo, Japan) coupled with Q-Exactive (Thermo Fisher Scientific, Waltham, MA, USA). The peptides were separated by nano-LC on an in-house packed Ultimate XB-C18 column (3 μm, 12 × 75 μm, Welch Materials, Ellicott, MD, USA) at a flow rate of 300 nL/min. Each fraction was dissolved in 0.1% FA, then injected and eluted using a gradient of 5%–30% solvent B (95% acetonitrile, 0.1% FA) over 40 min. The mass spectrometers were operated in a data-dependent mode, automatically switching between MS and MS2 acquisition. Survey full scan MS spectra (*m*/*z* 350–1800) were acquired in the Orbitrap with resolution 70,000. The 20 most intense ions were sequentially isolated and fragmented by high erenergy collisional dissociation (HCD). Peptides with unassigned charge states as well as with charge states less than +2 or more than +6 were excluded from fragmentation. Fragment spectra were recorded in the Orbitrap mass analyzer with resolution 17,500. The dynamic exclusion was enabled with repeat count 2 and exclusion duration of 8 s.

### 3.3. RNA-Seq

Total RNA from the spines was extracted with Trizol reagent (Invitrogen, Carlsbad, CA, USA) and purified using RNeasy Animal Mini Kit (Qiagen, Valencia, CA, USA). The construction of cDNA libraries and RNA-seq were performed as previously reported [1,3]. In brief, the poly-A containing mRNA molecules was purified using poly-T oligo-attached magnetic beads. Subsequently, the mRNAs were fragmented into small pieces using divalent cations under elevated temperature. The cleaved RNA fragments were copied into first strand cDNA using reverse transcriptase and random primers, and the second strand cDNA was synthesized using DNA polymerase I and RNaseH (Takara Biotechnology, Beijing, China). These cDNA fragments were ligated with the adapters, and these products were then purified and enriched with PCR to create the final cDNA libraries. Finally, the generated cDNA libraries were sequenced through Illumina HiSeq™ 2000 system at BGI-tech (Shenzhen, China).

### 3.4. RT-PCR

Total RNA was prepared as described above. cDNA was reverse transcribed from 2000 ng of DNase-treated total RNA extracted from venom glands, using the M-MuLV First Strand cDNA Synthesis Kit (Sangon, Shanghai, China). Primary RT-PCR reactions were performed in volumes of 50 μL containing 0.5 μL of cDNA (1000 ng), 0.5 μL of rTaq DNA Polymerase (Takara Biotechnology), 1× PCR reaction buffer (Takara Biotechnology), 200 μM of each dNTP, 0.2 μM of forward and reverse oligonucleotides (Supplementary Table S1). To confirm the quality of cDNA, the PCR was performed using the primers of house-keeping gene β-Actin. All PCR amplicons were analyzed by gel electrophoresis and subsequent sequencing to verify these predicted toxin sequences.

## 4. Conclusions

It is the first report to predict toxin genes for the venom gland of Chinese yellow catfish by combination of transcriptomic and proteomic sequencing. The combined analysis proved that the venom contains various toxins, at least including Veficolin, Ink Toxin, Adamalysin, ZaGP and CRISP.

Our findings suggest that the NGS coupled with LC-MS/MS is an effective method for the research of the transcriptome for those species lacking reference genome sequences. The use of transcriptomics and peptidomics approaches has provided an opportunity for getting a quick survey of fish venoms and has permitted the identification of toxin proteins/peptides. Therefore, to a certain degree, the pipeline combined SignalP, HMM and proteomics is also useful for the toxin prediction for other venom fish, especially for discovering homologues in distant species. This will allow us (1) to study the diversification of venom genes across various fish taxa; (2) to compare parallel and adaptive evolution of venom genes in distinct branches of the fish tree; and (3) to use the data to reassess the evolutionary history and phylogenetic relationships among the various venomous fish species, which may provide valuable insights in novel drug development and the recent spread of toxic animals across various ecosystems (e.g., lion fish invasions in the Atlantic Ocean).

## Figures and Tables

**Table 1:** Summary of sequence numbers in our achieved Toxin Database.

Group of Species	Taxonomy Name	Numbers of Sequences
Snakes	Serpents	1406
Scorpions	Scorpions	1510
Spiders	Araneae	1047
Cone snails	Conus	3860
Sea anemones	Actiniaria	308
Insects	Hexapoda	162
Fish	Teleostei	31
Mammals	Mammalias	106
Lizards	Heloderma	241
Jellyfish	Cubomedusae/Scyphozoa	175
Sea stars	Asteroidea	8
Hydra	Hydroida	14
Worms	Cerebratulus	5
Forg ,Toad	Amphibia	64
Sea-urchin	Echinoidea	2
Sea hare	Aplysia	22
	Annelida	11
Scolopendra	Myriapoda	9
All	Metazoa	8863

## Supplementary Materials

Supplementary materials can be found at http://www.mdpi.com/1422-0067/17/4/556/s1.
