# Supplementary material for: Prediction of Toxin Genes from Chinese Yellow Catfish Based on Transcriptomic and Proteomic Sequencing
Source: Int J Mol Sci. 2016 Apr 13;17(4):556. doi: 10.3390/ijms17040556 (PMC4849012; doi:10.3390/ijms17040556)
Supplement: Supplementary file 1 [file ijms-17-00556-s001.zip › ijms-124823-Supplementary materials/ijms-124823-Supplementary Materials.pdf]

Supplementary Materials: Prediction of Toxin Genes from Chinese Yellow Catfish Based on Transcriptomic and Proteomic Sequencing

Bing Xie, Xiaofeng Li, Zhilong Lin, Zhiqiang Ruan, Min Wang, Jie Liu, Ting Tong, Jia Li, Yu Huang, Bo Wen, Ying Sun and Qiong Shi

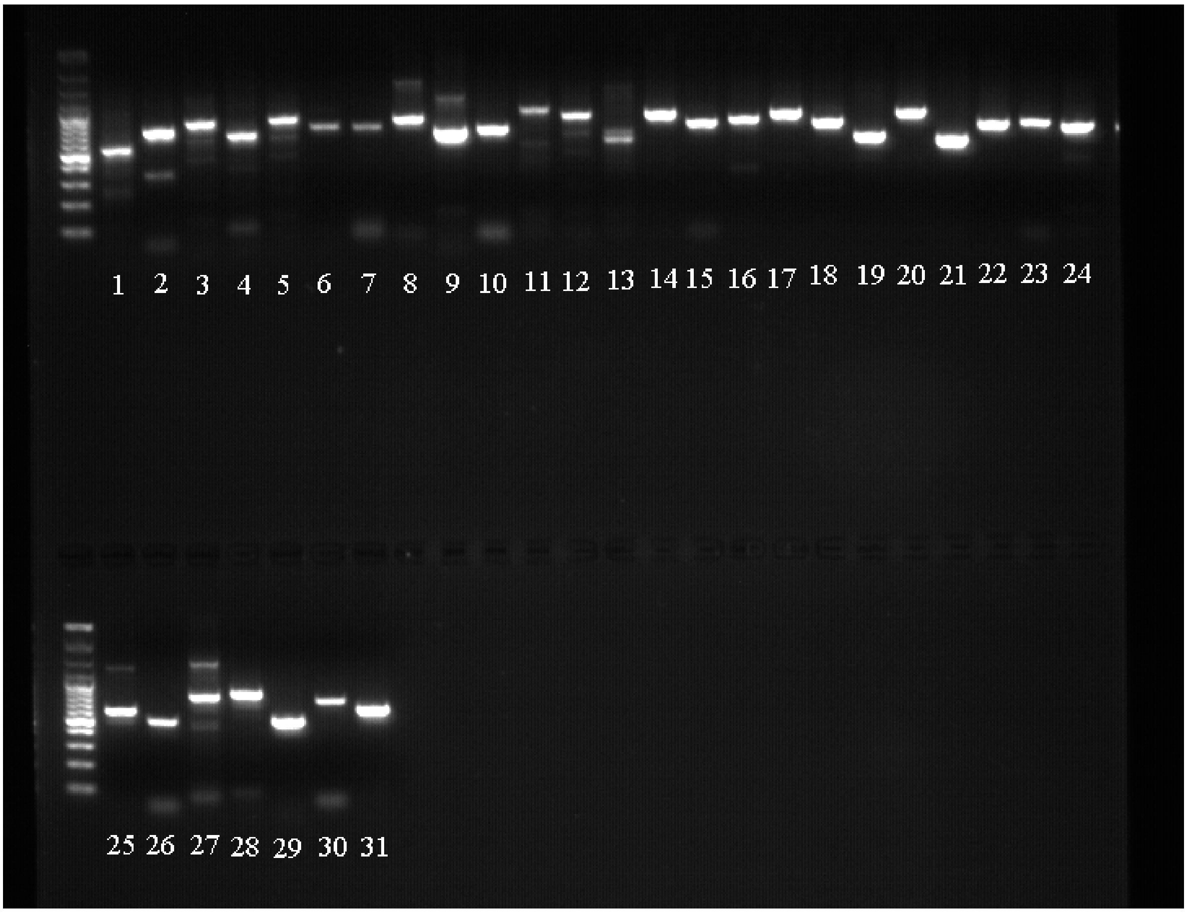

Figure S1. Gene amplification from putative toxins.
